# The cognitive chronometric architecture of reading aloud: semantic and lexical effects on naming onset and duration

**DOI:** 10.3389/fnhum.2012.00287

**Published:** 2012-10-19

**Authors:** Layla Gould, Jacqueline Cummine, Ron Borowsky

**Affiliations:** ^1^Department of Psychology, University of SaskatoonSaskatoon, SK, Canada; ^2^Department of Speech Pathology and Audiology, University of AlbertaEdmonton, AB, Canada

**Keywords:** reading aloud, semantic processing, lexical processing, naming response onset, naming response duration, word frequency, semantic neighborhood density, additive factors method

## Abstract

We examined onset reaction time (RT) in a word naming task using an additive factors method (AFM). The pattern of additive and over-additive joint effects on RT among Instructions (INST: name all, name words), Word Frequency (WF: log_10_ HAL), Semantic Neighborhood Density (SND: Inverse Ncount), and Word Type (WT: regular, exception) supported a cognitive chronometric architecture consisting of at least two cascaded stages of processing, with the orthographic lexical system as the locus of the INST × WF and the INST × SND interactions, and the phonological output system as the locus of the WF × WT and the SND × WT interactions. Additivity between INST and WT supports the notion that these variables affect separable systems, and a WF × SND interaction supports a common locus of their effects. These results support stage-like/cascaded processing models over parallel processing models of basic reading. We also examined response duration (RD) in these data by recording and hand-marking vocal responses, which provides evidence that basic reading processes are ongoing even after the initiation of a vocal response, and supports the notion that the more lexically a word is read, the shorter the RD. As such, the effects of WT and INST on RD were opposite to their effects on RT however the effects of WF and SND on RD were in the same direction as their effects on RT. Given the combination of consistent and dissociating effects between RT and RD, these results provide new challenges to all models of basic reading processes.

Semantic knowledge represents our worldly understanding of what things mean, how to interact with objects in our environment, how to interpret symbols and actions, as well as the meanings of words. As such, semantic knowledge is core to understanding not only language, but to understanding perception and cognition, and our world, in general. Although many years have been devoted to studying semantic knowledge, this concept has been a difficult one to elucidate due to its breadth. There are numerous ways to operationalize semantic processing, which provides multiple perspectives on the issue, but also broadens the problem space as opposed to narrowing it. However, as researchers have focused on and operationalized particular aspects of semantic knowledge, some substantial progress has been made (e.g., Balota et al., 2006; Yap et al., 2011).

Yap et al. (2012) recently demonstrated that semantic variables such as semantic neighborhood density (SND), number of features, semantic ambiguity (i.e., number of senses), imageability, and body-object interaction were reliable predictors of performance in several tasks of lexical processing. The only exceptions were the effects of SND and semantic ambiguity in the speeded pronunciation task. The null effect of semantic ambiguity in pronunciation has previously been argued to represent a lack of semantic influence in naming compared to lexical decision, for which there is an advantage for words with multiple meanings (Borowsky and Masson, 1996). Borowsky and Masson argued that the lexical decision task involves a monitoring of activation in orthographic, phonological, and semantic systems, thereby allowing for a familiarity-based lexical decision to benefit from multiple semantic representations (see also Balota and Chumbley, 1984; Chumbley and Balota, 1984), whereas naming can be accomplished without involvement of semantics and thus the lesser effect of semantic ambiguity in naming. It is possible that the effects of SND may behave similarly to the effects of semantic ambiguity, in that there may be an advantage for higher SND under conditions that encourage lexical access (see also Balota et al., 2004; Yap and Balota, 2009). One of the goals of the present research is to explore word naming behavior under conditions where lexical access is either compulsory or not. Another goal is to expand the investigation of naming behavior to more than just the onset of response, as has been done by Balota et al. (1989). Balota et al. explored duration of vocalizations in a semantic priming paradigm, similar to Balota and colleagues' work with other basic reading tasks involving parameters beyond response onset (e.g., Abrams and Balota, 1991; Bangert et al., 2012). As a general principle, going beyond the initial onset of response provides a larger window through which to view the effects of underlying cognitive processes. As perhaps the most ecologically valid basic reading task, the task of reading aloud is critical to explore in terms of both of our goals of manipulating lexical/semantic access and examining both response onset and duration.

## Methodological considerations in reading aloud

The measurement of vocal onset *reaction time* (RT) has been central to research on basic cognitive processes since the invention of the voice-key (Dunlap, 1913; Boder, 1933). Although many researchers had initially assumed that the initiation of a vocal response first requires the generation of a complete phonological code for the entire word, this assumption has been challenged in recent years (e.g., Hudson and Bergman, 1985; cf. Rastle et al., 2000). Furthermore, research involving a delayed naming task (i.e., pronunciation is delayed until a cue is given) has demonstrated that the frequency effect still manifests in onset RT even after delays up to 1400 ms (Balota and Chumbley, 1985; see also Monsell et al., 1989). As such, it appears that the influences of lexical variables such as word frequency (WF) are still having an effect even after sufficient time to prepare and initiate a response. Delayed naming evidence notwithstanding, it is unclear why it would be necessary to hold off the initiation of the vocal response until the entire word is decoded, especially given the typical instructions (INST) to name words as quickly and accurately as possible. Furthermore, several models of reading refer to: (1) a relatively slow serial grapheme-to-phoneme translation system, which allows for the naming of novel words in a serial/cascaded fashion, as well as (2) a relatively fast lexical system, which allows words to be named in a “whole-word” manner (e.g., Coltheart et al., 2001; Coltheart, 2006; Borowsky et al., 2012). Nearly a century of research based on vocal onset RTs has been conducted to explore these and other basic reading processes. Given that cognitive processes could be operating beyond the initiation of vocal onset, it is important to explore measures of naming responses that go beyond measuring the onset. Another major goal of our present research involves exploring the *response duration* (RD) of vocal responses in addition to RT.

Research into the chronometric architecture of cognition also has a long history. Donders' (1969) subtractive logic provided the first method of examining when certain cognitive processes were occurring. For example, if one were to subtract the time that it takes to respond to the presence or absence of a flash of light, from the time that it takes to respond to a flash of light of a certain color, one could attribute the difference in time to color processing. However, this logic requires the untenable assumption of *pure insertion*, whereby more than just color processing has been inserted into the task (e.g., holding in memory the instructed target color). Sternberg (1969) argued that *pure insertion* was not a tenable assumption, and developed the Additive Factors Method (AFM) (see also: Borowsky and Besner, 1993; Roberts and Sternberg, 1993; Stolz and Neely, 1995; Yap and Balota, 2007). By looking at the joint effects of the variables, this method allows for the examination of whether two variables are affecting the same system in time (i.e., over-additive interactive effects on RT) or separable systems in time (i.e., additive effects on RT). Another major goal of our present research involves exploring the joint effects of four variables that are known to reflect the operation of subsystems of basic reading processes: SND, WF, WT, and INST.

## Effects that reflect the operation of lexical/semantic subsystems

**Figure 1:**
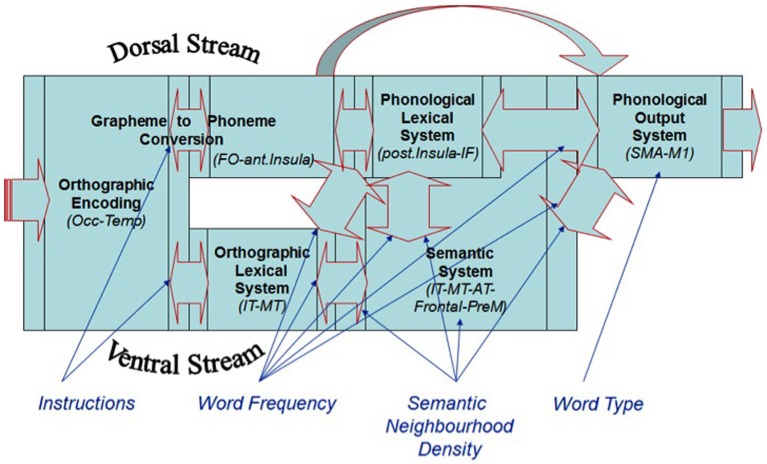
**A dual-stream, ventral-lexical, dorsal-sublexical, cascaded processing framework for basic reading processes**.

As described earlier, semantic knowledge is core to any model of language processing. SND has been shown to be a measure of semantic processing (Shaoul and Westbury, 2010). This measure reflects the number of words that co-occurred with a target word within a fixed distance threshold, as determined by an analysis of 57,153 words present in Wikipedia in April 2010 (a total of 971,819,808 occurrences). Words that have a large number of semantic neighbors show benefits relative to words that have a small number of semantic neighbors, as was shown by Yap et al. (2012) using the tasks of: lexical decision, go/no-go lexical decision, speeded naming, progressive demasking, and semantic classification. SND could serve to facilitate semantic processing, as well as connections to other word-level systems such as the orthographic lexical system and the phonological output system, in that the higher the SND the higher the number of facilitative connections both within and between levels (as is typical of interactive activation architectures, McClelland and Rumelhart, 1981; Coltheart, 2006; Yap et al., 2012; see Figure 1).

There are several models that propose that printed WF effects manifest in the lexical/semantic systems (e.g., Morton, 1979; McClelland and Rumelhart, 1981; McCann and Besner, 1987; Borowsky and Besner, 1993; Reynolds and Besner, 2005). For example, WF could affect the connections between the lexical subsystems and semantic system, whereby the more frequently a word is read, the faster the rate of activation in these systems, and the faster the RT (see Figure 1; Borowsky and Besner, 1993). The WT [i.e., regular vs. exception words (EXCs)] effect on RT is another effect that reflects basic reading processes. Given that regular words (REGs) can be pronounced correctly through both the sublexical grapheme-to-phoneme conversion (GPC) route (allowing the word to be “sounded out”) or the orthographic lexical route (allowing the word to be read in a “whole-word” manner), these routes produce the same pronunciation at the phonological output system. Conversely, EXCs must be processed via the orthographic lexical route to be pronounced correctly. EXCs produce a slower RT because the two routes produce conflicting pronunciations, and a single phonological output must ultimately be selected, particularly in the case of low frequency EXCs.

WT has also been found to interact with WF on naming RT, whereby EXCs produce slower RTs and elicit a greater WF effect, compared to REGs (e.g., Monsell et al., 1992; Cummine et al., 2010). This same interactive pattern has been shown on the Blood Oxygenation Level Dependent (BOLD) function intensity in the supplementary motor area (SMA), which likely represents the phonological output system given that the SMA is the last cortical region prior to activating the motor cortex (see Figure 1; Cummine et al., 2010). Other reading route reliance effects have also been reported, whereby there is flexibility on route reliance depending on stimulus and task manipulations (e.g., Rastle and Coltheart, 1999; Zevin and Balota, 2000; Borowsky et al., 2002; Reynolds and Besner, 2005; see Balota et al., 2006 for a review). Given the proximity of SND effects to WF effects in the basic reading architecture, in that they both involve lexical/semantic systems, it seems reasonable that SND and WF should also interact due to these common influences.

Researchers have begun to explore the strategic effects of INST on reading. For example, Hino and Lupker (2000, see also Kinoshita and Woollams, 2002) presented participants with a list of words and non-words (NWs), and used what we refer to as a *name words* condition and a *name all* condition. INST to *name words* required the participant to name a stimulus aloud only if it spells a word, which forces the participant to process the word via the orthographic lexical route, as they must first verify that the stimulus is in fact a word (see Figure 1). Cummine et al. (2012) provided direct functional and behavioral evidence that INST to *name words* forces reliance on the orthographic-lexical route. We reported that INST to *name words* showed greater visible activation along the ventral-lexical stream, as well as produced greater WF effects on RT relative to INST to *name all* stimuli. There was no interaction between INST and WT (i.e., additivity), but there was an interaction between WF and WT under the normal *name all* instruction condition. When the AFM is employed, additive and interactive joint effects can reveal the loci of effects among the processing systems, and how many systems are involved in the cognitive chronometric architecture.

## Additive factors method

**Figure 2:**
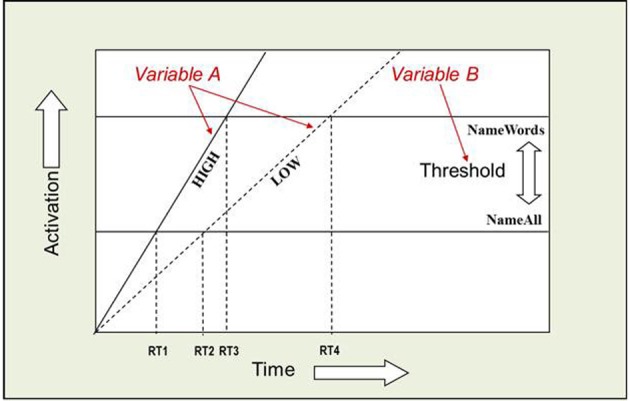
**Over-additive interaction between INST and WF.** An Additive Factors Method interpretation of this interaction is that both INST and WF are affecting a common system in time. If INST are assumed to affect the threshold for activation, and WF is assumed to affect the rate of activation over time, or vice versa, then the points in time when each rate crosses a threshold correspond to the average onset RTs. [(RT4 − RT2) > (RT3 − RT1)] and [((RT2 + RT4)/2) > ((RT1 + RT3)/2)]. WF and WT interact in a similar fashion.

**Figure 3:**
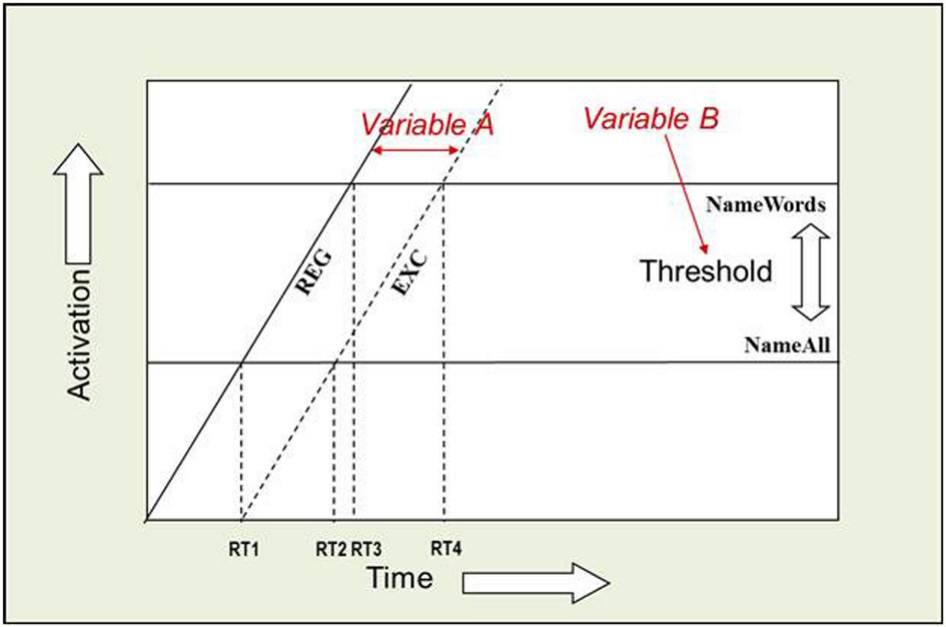
**Additive joint effects between INST and WT.** An Additive Factors Method interpretation of this additive effect is that INST and WT are affecting separable systems in time. If INST are assumed to affect the threshold for activation (i.e., the amount of time it takes to verify that a letter string spells a word), and WT is assumed to shift the rate of activation over time (i.e., the time it takes to choose among the competing phonological codes for EXCs), or vice versa, then the points in time when each rate crosses a threshold correspond to the average onset RTs. [(RT4 − RT2) = (RT3 − RT1)] and [((RT2 + RT4)/2) > ((RT1 + RT3)/2)].

**Figure 4:**
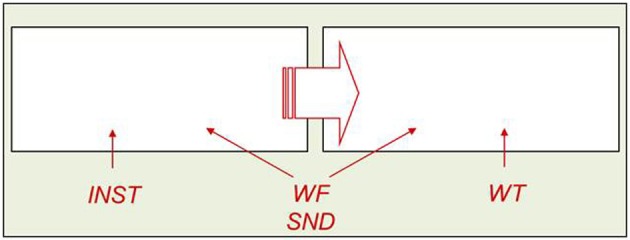
**A cognitive chronometric architecture to account for the INST × WF, INST × SND, WF × WT, and SND × WT over-additive interactions, and INST + WT additivity**.

The AFM proposes that if two variables interact over-additively on RT (such as the WF × WT interaction described above), it is indicative of those variables affecting a common system of processing in time (see Figure 2). Also, the over-additive interaction between INST and WF is indicative of these two variables affecting a common system (Cummine et al., 2012). In contrast, if two variables produce additive effects on RT, those variables are assumed to be affecting separable (even if they are cascaded; McClelland, 1979) systems of processing (see Figure 3). As such, the additive pattern found between INST and WT is taken to indicate that those variables are affecting separable systems of processing. Taken together, these joint effects support a cognitive chronometric architecture of at least two systems (see Figure 4), whereby INST and WF interact in a relatively early system that serves to gate the processing of words through the orthographic lexical route when the INST are to *name words* only (resulting in a lower threshold, see Figures 2 and 3), and WF and WT interact in the relatively late phonological output system in a similar fashion. The present research examines SND in addition to these variables in order to further constrain the architecture for basic reading processes. Given that WF and SND affect some common systems and connections, one would expect that SND should show similar joint effects with INST and WT as did WF (see Figures 1 and 4).

## Naming response duration

As previously mentioned, one major goal of our present research involves not only exploring measures of onset RT, but also the RD of vocalizations. Previous research on naming responses has almost solely relied on voice-key measures of *onset* RT. However, given that basic reading processes may still be operating while initiating a vocal response, onset RT measures may not be comprehensive enough. Examining the *duration* of vocal responses should serve as an important additional measure of word processing. Balota et al. (1989) showed that RD is an important converging measure of reading processing in that naming RDs are significantly shorter when a related word is presented. Given that their relatedness manipulation served to decrease RD, this supports the notion that manipulations that enhance lexical/semantic processing yield shorter RDs. Thus, one can predict that shorter RDs should reflect lexical “whole-word” reading, whereas longer RDs should reflect sublexical GPCs. Consistent with the view that RDs can reflect important aspects of cognitive processing post-onset RT, Kawamoto et al. (1999) research on onset and rime durations suggests that the criterion to initiate pronunciation is based on the initial phoneme and not the whole-word. That said, RD effects could also reflect how familiar we are with a given word's pronunciation, whereby the more often we pronounce a given word, the shorter the RD could get as a function of the word being read more lexically over time.

Our present research also contributes a novel means of measuring RT and RD in word recognition, whereby one manually analyzes speech envelopes of verbal responses to objectively measure the onset and offset of a naming response. Previous studies have found that using voice-keys to measure onset RT may be quite unreliable. For example, a study by Rastle and Davis (2002) found that different types of voice-keys can produce different results, whereby the voice-keys were often triggered at different points in time following the actual onset of the naming response. They found that hand-marking the acoustic onset of each word using visual waveforms of intensity over time can produce less error than voice-keys, and thus suggest that visually investigating these waveforms may be an important way to check voice-key onsets. We also note that the proportion of errors that are due to voice-key problems can be quite substantial (e.g., Balota et al.'s, 2007, large scale study of naming reported that nearly 13% of naming errors were due to voice-key problems). Our labs has begun to digitally record participants' vocal responses and then manually inspect each vocal response using PRAAT digital software (Boersma and Weenink, 2012). By using both visual and auditory cues to identify vocalization onset and offset, it allows us to precisely measure the onset RT and RD of each response. Given the potential for variability of voice-key measurements, in the present experiments we analyze the RTs obtained via hand-marking (Experiment 1), and RTs obtained via a voice-key (Experiment 2). Importantly, we also measure the RDs via hand-marking in both experiments, which has not been previously reported.

## Hypotheses

Our first set of hypotheses involves the joint effects of INST, WF, SND, and WT on onset RT (see Figure 1). We predict that: (1) *INST × WF*—to the extent that INST and WF are both affecting the orthographic lexical system, they should show an over-additive interaction on onset RT; (2) *INST + WT*—because we are predicting that INST are having their effect early by gating processing towards the orthographic lexical system under *name words* INST, whereas WT has been shown to have its effect later at the phonological output system, these two variables should show an additive pattern on RT; (3) *INST × SND*—to the extent that INST and SND are both affecting the orthographic lexical system, they should show an over-additive interaction on onset RT; (4) *WF × SND*—to the extent that WF and SND are affecting the semantic system and connections to other word-level systems, they should also show an over-additive interaction on onset RT; (5) *SND × WT*—to the extent that SND and WT are affecting the phonological output system, they should also show an over-additive interaction on onset RT; and (6) *WF × WT*—to the extent that WF and WT are affecting the phonological output system, they should produce the typical over-additive interaction under normal *name all* INST.

Our second set of hypotheses are in regards to the RDs of vocal responses: *EXC RD* < *REG RD < NW RD*—given that EXCs must be processed as whole-words and read via the relatively fast lexical system in order to be pronounced correctly, they should produce the shortest RDs, despite the fact that EXC onset RTs are longer than REG onset RTs. Given that NWs must be processed through the relatively slow sublexical GPC system, they should produce the longest RDs. Finally, given that REGs can be processed through either route, they should elicit intermediate RDs relative to EXCs and NWs, despite having the fastest RTs^1^. With respect to INST, given that the *name words* condition also forces participants to rely on the orthographic lexical system, it should produce shorter RDs compared to the *name all* condition (*name words RD < name all RD*). Given Balota et al.'s demonstration of semantic priming having a facilitative effect on naming onset RT and naming RD, the SND effect should remain in the same facilitative direction for onset RT and RD in the present experiment. Furthermore, given that WF is considered to have its effects at the same semantic/lexical level as SND, the WF effect should also remain in the same facilitative direction for onset RT and RD.

In the experiments that follow, Experiment 1 (*n* = 20) was conducted in a MRI scanner (see Cummine et al., 2012), and Experiment 2 (*n* = 40) was conducted in a behavioral lab. Although the results of these experiments are presented separately, we will focus our discussion on analyses that combine the data from Experiments 1 and 2.

## Experiment 1

### Methods

#### Participants

Twenty participants responded to a local advertisement for a fMRI experiment at the University of Alberta (see Cummine et al., 2012 for details). The experiment was performed in compliance with the relevant laws and institutional guidelines, and was approved by the University of Alberta Health Research Ethics Board. The participants' consent was obtained according to the Declaration of Helsinki (1996). Inclusion criteria consisted of normal or corrected-to-normal vision and English as a first language. Eighteen participants were right-handed.

#### Stimuli

One hundred and twenty-six pairs of monosyllabic REGs and EXCs matched for initial onset and length were used as critical stimuli (Patterson and Hodges, 1992). SND, as described earlier in the introduction, was measured using inverse Ncount (Shaoul and Westbury, 2010), which is the inverse of the number of semantic neighbors +1. These stimuli were well-matched on several of the characteristics available from the E-Lexicon Database (http://elexicon.wustl.edu/, Balota et al., 2007), as we found that WT (REG = 0, EXC = 1) did not correlate significantly with log_10_ HAL WF (*r* = 0.036, *p* = 0.57), bigram frequency by position (*r* = 0.033, *p* = 0.60), bigram mean frequency, (*r* = −0.051, *p* = 0.42), bigram sum frequency (*r* = −0.036, *p* = 0.57), number of morphemes (*r* = 0.058, *p* = 0.357), number of phonemes (*r* = −0.082, *p* = 0.20), phonological neighborhood (*r* = 0.081, *p* = 0.202), or inverse Ncount (*r* = −0.031, *p* = 0.50). These words can be considered to be of fairly high familiarity, as their mean WF is relatively high (log_10_ HAL WF mean = 9.63). A set of 128 pronounceable NWs were also generated from the critical words by changing one or two letters. The mean length of the NWs (4.48 letters) was well matched to the mean length of the words (4.51 letters for both the EXCs and REGs), [*t*_(252)_ = 0.307, *p* = 0.759]. For each INST condition, a total of 190 stimuli were presented in two blocks (one block had 31 REGs, 32 EXCs, and 32 NWs, and the other block had 32 REGs, 31 EXCs, and 32 NWs), such that every participant was presented with each stimulus only once, and stimuli were cycled through INST conditions across participants so that each stimulus was presented equally often under each INST set.

#### Procedure and apparatus

For the *name all* INST condition, participants were instructed to “read aloud each letter string, as quickly and accurately as possible.” For the *name words* INST condition, participants were instructed to “only read aloud each letter string that spells a word, as quickly and accurately as possible.” Letter strings were presented, and participants responded vocally, during a regular periodic gap in the image acquisition that followed the offset of each volume of images (i.e., a sparse-sampling, or gap, paradigm; Borowsky et al., 2005a,b, 2006, 2007, 2012; Cummine et al., 2010, 2012). That is, a letter string was presented for 1850 ms during a silent gap, at the offset of a 1850 ms acquisition of a volume of images, allowing participants to name aloud the letter string immediately and without gradient noise in the background. Letter strings were randomly selected, without repetition, and back-projected one at a time on a screen such that they were visible to the participants through the mirror on the head coil. A computer running EPrime software (Psychology Software Tools, Inc., http://www.pstnet.com) was used to trigger each image acquisition in synchrony with the presentation of visual stimuli.

**Figure 5:**
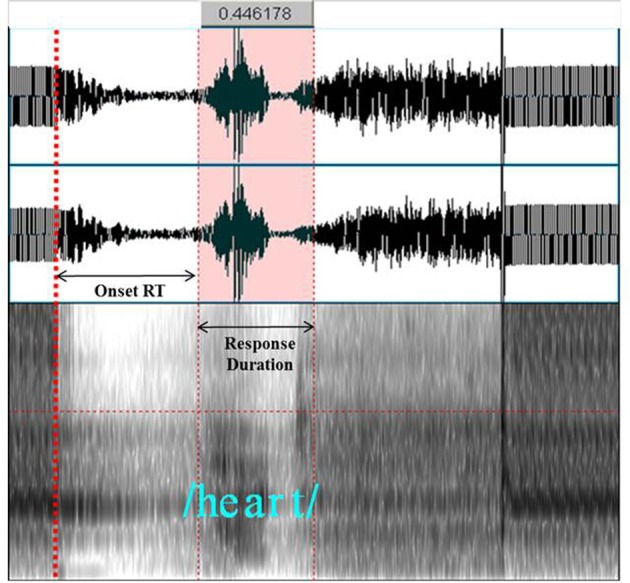
**An example of using PRAAT to assist in localizing the onset RT and RD of the vocalization “heart.”** Offset of gradient noise from the MRI can be seen and heard at the time point of the coarse red-dashed line (relevant for Experiment 1 only). The onset and offset of the vocalization can be seen and heard between the thin red-dashed lines, while the temporal distance between those lines (i.e., the RD) is indicated at the top. Visual inspection of both the spectrograms and the formants, as well as several replayings of the audio recording, allowed for precise measurement of the onset RT and RD.

Vocal responses were recorded at 96 KHz, 24 bit, through the intercom using an Olympus LS11 digital recorder, during the acquisition gap. These recordings were then analyzed using PRAAT software (Boersma and Weenink, 2012), and the speech spectrograms and formants were used to localize vocalization onset RT and the RD. Given that the gradient noise associated with the final image acquisition in each volume coincided with the onset of the target stimulus, we were able to use it as an auditory and visual cue on the digital recording for identifying when the stimulus appeared on the screen (see Figure 5). By replaying the audio recording, we were able to code whether each participant's response was correct, incorrect, or a spoiled trial. By using PRAAT to analyze the speech spectrograms, and by replaying the audio recording, we were able to determine the exact time point for the onset RT and the RD.

### Results

#### Word naming reaction time

**Figure 6:**
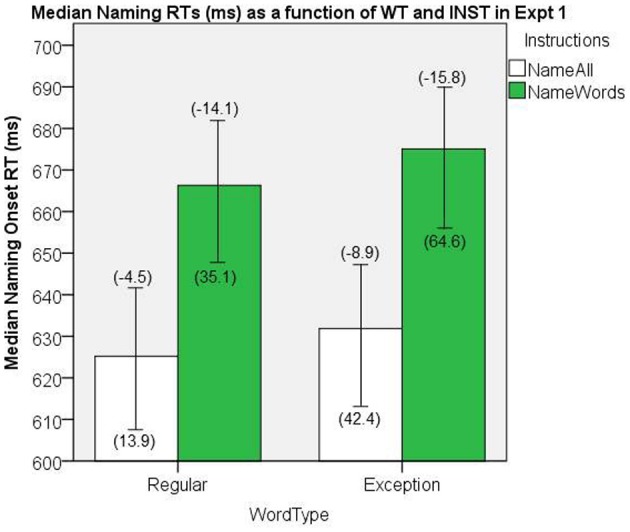
**Median Naming RTs (in ms) as a function of WT and INST in Experiment 1.** The 95% C.I.s are presented as error bars using Loftus and Masson, (1994) method. Co-efficients relating WF to RT (ms/log_10_HALWF) are presented in parentheses above each error bar, and co-efficients relating SND to RT (ms/unit inverseNcount) are presented in parentheses below each error bar.

The naming onset RT data were first aggregated by participant as a function of INST (*name all, name words*) and WT (REG and EXC). Medians of the correctly named item RTs were submitted to a 2 × 2 general linear model (GLM) ANOVA, with WT and INST as repeated measures factors. The median naming onset RTs are presented in Figure 6. There were significant main effects of INST, [*F*_(1, 19)_ = 7.626, *MS*e = 4659, *p* = 0.01], and WT, [*F*_(1, 19)_ = 4.97, *MS*e = 239, *p* = 0.04], and no significant interaction, [*F*_(1, 19)_ = 0.049, *MS*e = 442, *p* = 0.83].

#### Non-word naming reaction time

The NW condition in the *name all* INST condition yielded a median onset RT of 687.7 ms (Loftus and Masson, (1994), repeated-measure 95% confidence interval (CI) = ± 21.9).

#### Accuracy

The mean accuracy rates resulted in 100% accuracy in all cells, and thus there was no variance for a statistical analysis.

#### Word frequency effects on reaction time

In order to evaluate the effects of WF as a continuous variable, GLM regressions were conducted on each participant's correct onset RTs, with RT as the dependent variable, and WF as a continuous independent variable, separately for each combination of INST and WT. The resulting WF coefficients for each INST and WT set were then aggregated over participants (e.g., Borowsky et al., 2002), and submitted to a 2 × 2 GLM ANOVA, with WT and INST as repeated measures factors. This analysis allows one to generalize to both items and participants, in that items are treated as the unit of analysis in the regressions, and that participants are treated as the unit of analysis when the co-efficients are being statistically tested. Given our use of the AFM and a focus on two-way joint effects, interaction effects were restricted to two-way joint effects in all the analyses reported here. Figure 6 shows the mean co-efficients above the median RTs. There was a significant main effect of INST on the size of WF effect, [*F*_(1, 19)_ = 15.5, *MS*e = 83.6, *p* = 0.001], which represents an INST by WF interaction on naming RT, whereby the WF effects are greater under *name words* INST. The main effect of WT on the size of WF effect was not significant, [*F*_(1, 19)_ = 1.38, *MS*e = 115.2, *p* = 0.25]. An analysis of this WF by WT interaction for *name all* INST failed to show a significant effect, [*t*_(19)_ = −1.27, *SEM* = 3.47, *p* = 0.22].

#### Semantic neighborhood density effects on reaction time

In order to evaluate the effects of SND as a continuous variable, GLM regressions were conducted on each participant's correct onset RTs, with RT as the dependent variable, and SND as a continuous independent variable, separately for each combination of INST and WT. The resulting SND co-efficients for each INST and WT set were then aggregated over participants, and submitted to a 2 × 2 GLM ANOVA, with WT and INST as repeated measures factors. Figure 6 shows the mean co-efficients below the median RTs. There was a significant main effect of INST on the size of the SND effect, [*F*_(1, 19)_ = 5.53, *MS*e = 1698.7, *p* = 0.03], which represents an INST by SND over-additive interaction on naming RT, whereby the SND effects are greater under *name words* INST. There was also a significant main effect of WT on the size of the SND effect, [*F*_(1, 19)_ = 8.69, *MS*e = 1938.8, *p* = 0.008], which represents a WT by SND over-additive interaction on naming RT, indicating that the SND effect is greater for EXCs than REGs.

#### Word naming response duration

**Figure 7:**
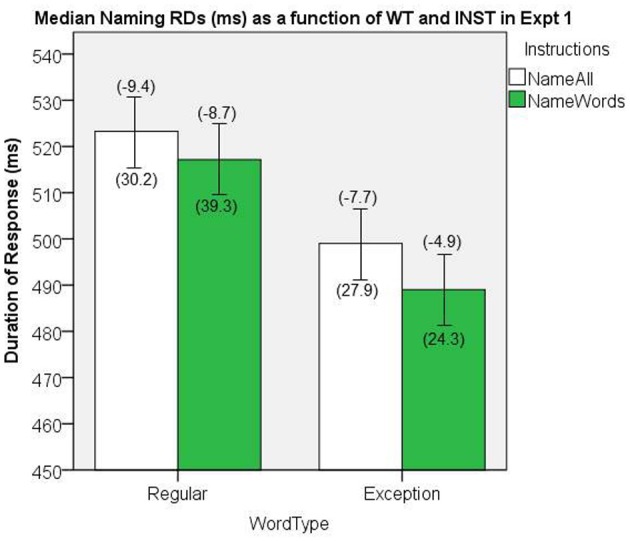
**Median Naming RDs (in ms) as a function of WT and INST in Experiment 1.** The 95% C.I.s are presented as error bars using Loftus and Masson, (1994) method. Co-efficients relating WF to RT (ms/log_10_HALWF) are presented in parentheses above each error bar, and co-efficients relating SND to RT (ms/unit inverseNcount) are presented in parentheses below each error bar.

The naming RD data were aggregated by participant as a function of INST and WT. Medians of the correctly named item RDs were submitted to a 2 × 2 GLM ANOVA, with WT and INST as repeated measures factors. The median naming RDs are presented in Figure 7. There was a significant main effect of WT, [*F*_(1, 19)_ = 152.06, *MS*e = 90.3, *p* < 0.001]. The main effect of INST showed a trend in the predicted direction but was not significant, [*F*_(1, 19)_ = 1.72, *MS*e = 749.25, *p* = 0.20], and no significant interaction, [*F*_(1, 19)_ = 0.710, *MS*e = 105.7, *p* = 0.41].

#### Non-word naming response duration

The NW condition in the *name all* INST condition yielded a median RD of 587.9 ms (Loftus and Masson, 1994, repeated-measure 95% CI = ± 10.78).

#### Word frequency effects on response duration

We conducted analyses of WF effects on RD in the same way as our analyses on RT. Figure 7 shows the mean co-efficients above the median RDs. There was a significant main effect of WT on the size of WF effect, [*F*_(1, 19)_ = 9.42, *MS*e = 16.1, *p* = 0.006], which represents a WT by WF interaction on naming RD, whereby the WF effects are greater for REGs. A main effect of INST on the size of WF effect approached significance, [*F*_(1, 19)_ = 3.67, *MS*e = 17.3, *p* = 0.07], whereby there was a tendency for an interaction, such that there were larger WF effects in the *name all* condition.

#### Semantic neighborhood density effects on response duration

We conducted analyses of SND effects on RD in the same way as our analyses on RT, and the mean co-efficients are shown below the median RDs in Figure 7. There was no significant main effect of INST on the size of the SND effect, [*F*_(1, 19)_ < 1, *MS*e = 706.2, *p* = 0.99], nor was there a significant main effect of WT on the size of the SND effect, [*F*_(1, 19)_ = 2.15, *MS*e = 880.0, *p* = 0.16], which suggests there were no interactions between INST and SND, or WT and SND on RD.

### Discussion

For onset RT there was a significant main effect of WT and INST, but no interaction. This additive pattern supports the notion of WT and INST affecting separable systems (see Figure 4). The onset RT analysis involving WF supports an over-additive interaction with INST. This pattern of interaction with WF supports the notion of INST affecting the same system as that affected by WF. Our analysis of the SND effect on onset RT showed an over-additive interaction between INST and SND, as well as between SND and WT. This pattern of interaction with SND supports the notion of INST and WT both affecting the same system as that affected by SND.

In keeping with our hypotheses regarding the RDs of vocal responses, the pattern of results supported: *EXC RD* < *REG RD < NW RD*—in that the main effect of WT was significant, and that the 95% CI for NWs did not overlap with any of the comparison cells. Furthermore, there was a trend for the *name words* INST condition to have shorter RDs than the *name all* INST condition.

Our analysis of the WF effect on RD revealed a very interesting pattern. Specifically, larger WF effects are associated with the longer RD cells (i.e., REGs), despite the fact that the opposite pattern was demonstrated for onset RT. As such, RD is shorter for the lexically read EXCs, which supports our hypotheses about shorter RDs being associated with lexically read items. Our analysis of the SND effect on RD showed no significant Two-Way interactions.

## Experiment 2

### Methods

#### Participants

Forty undergraduate students participated for course credit in their introductory psychology class. The experiment was performed in compliance with the relevant laws and institutional guidelines, and was approved by the University of Saskatchewan Research Ethics Board. Inclusion criteria consisted of normal or corrected-to-normal vision and fluency in English. Thirty eight participants were right-handed.

#### Stimuli, procedure, and apparatus

These were identical to Experiment 1, with the following exceptions: Testing was done in a sound attenuated behavioral lab, stimuli were presented on a 15” Samsung CRT monitor connected to a PC running EPrime software, participants initiated each trial by pressing a button on the PST serial response box, which was also connected to a microphone that was interfaced with the voice-key for detecting vocalization onsets. RD was measured in the same way as Experiment 1 (see Figure 5).

### Results

The same analyses were conducted as in Experiment 1. One participant elicited error rates that were in excess of three SDs below the mean of all participants, and was thus excluded from the analyses.

#### Word naming reaction time

**Figure 8:**
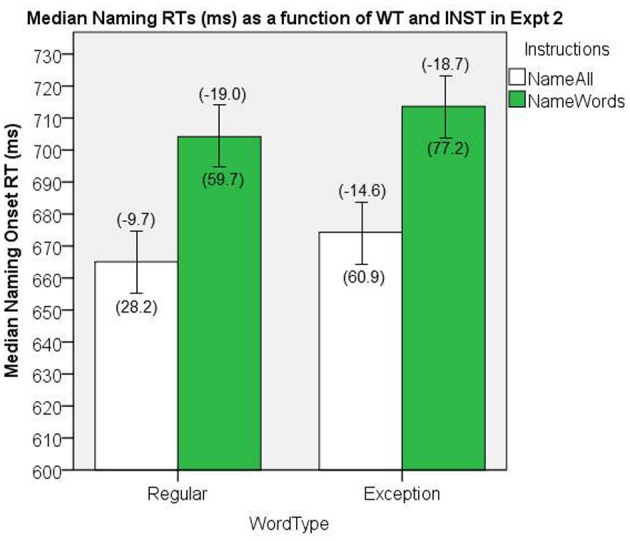
**Median Naming RTs (in ms) as a function of WT and INST in Experiment 2.** The 95% C.I.s are presented as error bars using Loftus and Masson, (1994) method. Co-efficients relating WF to RT (ms/log_10_HALWF) are presented in parentheses above each error bar, and co-efficients relating SND to RT (ms/unit inverseNcount) are presented in parentheses below each error bar.

The median naming onset RTs are presented in Figure 8. There were significant main effects of INST, [*F*_(1, 38)_ = 26.5, *MS*e = 2260.5, *p* < 0.001], and WT, [*F*_(1, 38)_ = 4.96, *MS*e = 686, *p* = 0.03], and no significant interaction, [*F*_(1, 38)_ = 0.463, *MS*e = 242, *p* = 0.96].

#### Non-word naming reaction time

The NW condition in the *name all* INST condition yielded a median onset RT of 750.8 ms (Loftus and Masson, 1994, repeated-measure 95% CI = ± 14.7).

#### Accuracy

**Figure 9:**
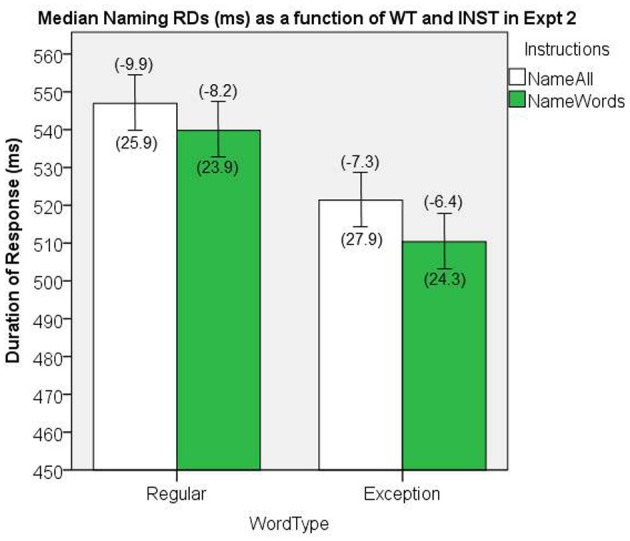
**Mean Proportion Accurate as a function of WT and INST in Experiment 2.** The 95% C.I.s are presented as error bars using Loftus and Masson, (1994) method.

The mean proportion accuracy rates are presented in Figure 9. There was a significant main effect of INST, [*F*_(1, 38)_ = 15.62, *MS*e = 0.003, *p* < 0.001], and WT, [*F*_(1, 38)_ = 11.44, *MS*e = 0. 001, *p* = 0.002], and there was no significant interaction, [*F*_(1, 38)_ = 2.64, *MS*e = 0.001, *p* = 0.11]. The NW accuracy in the *name all* INST condition yielded a mean proportion of 0.90 (Loftus and Masson, 1994, repeated-measure 95% CI = ±.017).

#### Word frequency effects on reaction time

We conducted analyses of WF effects on RT in the same way as our analyses in Experiment 1. Figure 8 shows the mean co-efficients above the median RTs. There was a significant main effect of INST on the size of the WF effect, [*F*_(1, 38)_ = 11.28, *MS*e = 154.7, *p* = 0.002], which represents a INST by WF interaction on naming RT, whereby the WF effects are greater for *name words* INST. The main effect of WT on the size of the WF effect (i.e., the WF by WT interaction) did not reach significance, [*F*_(1, 38)_ = 1.78, *MS*e = 114.1, *p* = 0.19], however, an analysis of this WF by WT interaction for the normal *name all* INST showed a significant effect, [*t*_(38)_ = −2.13, *SEM* = 2.30, *p* = 0.04].

#### Semantic neighborhood density effects on reaction time

We conducted analyses of SND effects on RT in the same way as our analyses in Experiment 1. Figure 8 shows the mean co-efficients below the median RTs. There was a significant main effect of INST on the size of the SND effect, [*F*_(1, 38)_ = 5.67, *MS*e = 3933.8, *p* = 0.02], which represents an INST × SND over-additive interaction, whereby the SND effect is larger for the *name words* INST condition. There was also a significant main effect of WT on the size of the SND effect, [*F*_(1, 38)_ = 4.46, *MS*e = 5534.5, *p* = 0.04], which represents a SND × WT over-additive interaction, whereby the SND effect is larger for EXCs than for REGs.

#### Word naming response duration

**Figure 10:**
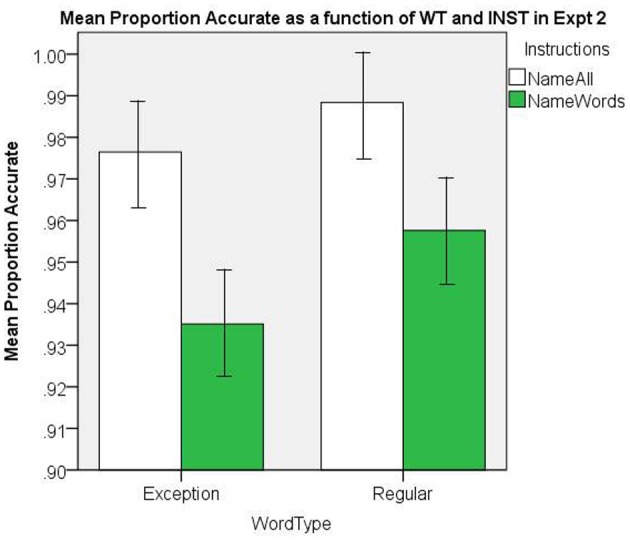
**Median Naming RDs (in ms) as a function of WT and INST in Experiment 2.** The 95% C.I.s are presented as error bars using Loftus and Masson, (1994) method. Co-efficients relating WF to RT (ms/log_10_HALWF) are presented in parentheses above each error bar, and co-efficients relating SND to RT (ms/unit inverseNcount) are presented in parentheses below each error bar.

The median naming RDs are presented in Figure 10. There was a significant main effect of WT, [*F*_(1, 38)_ = 140.29, *MS*e = 210.2, *p* < 0.001], which was in the predicted direction with EXCs showing shorter RDs. The main effect of INST approached significance, [*F*_(1, 38)_ = 2.7, *MS*e = 1187.8, *p* = 0.10], which was also in the predicted direction, and there was no significant interaction, [*F*_(1, 38)_ = 0.766, *MS*e = 186.3, *p* = 0.39].

#### Non-word naming response duration

The NW condition in the *name all* INST condition yielded a median RD of 561.5 ms (Loftus and Masson, 1994, repeated-measure 95% CI = ± 7.65).

#### Word frequency effects on response duration

We conducted analyses of WF effects on RD in the same way as our analyses in Experiment 1. Figure 10 shows the mean co-efficients above the median RDs. There was a significant main effect of WT on the size of the WF effect, [*F*_(1, 38)_ = 4.28, *MS*e = 45.2, *p* = 0.045], which represents a WT by WF interaction on naming RD, whereby the WF effects are greater for REGs. There was no main effect of INST on the size of WF effect, [*F*_(1, 38)_ = 1.42, *MS*e = 44.2, *p* = 0.24], although we note that the direction of the effects was consistent with Experiment 1.

#### Semantic neighborhood density effects on response duration

We conducted analyses of SND effects on RD in the same way as our analyses in Experiment 1. Figure 10 shows the mean co-efficients below the median RDs. There was no significant main effect of INST on the size of the SND effect, [*F*_(1, 38)_ = 0.17, *MS*e = 1826.0, *p* = 0.69], nor was there a significant main effect of WT on the size of the SND effect, [*F*_(1, 38)_ = 0.08, *MS*e = 652.1, *p* = 0.78].

### Discussion

Our first set of hypotheses involved the joint effects of INST, WF, SND, and WT on onset RT. Consistently in both Experiments 1 and 2, we showed that: (1) *INST × WF*—the over-additive INST × WF interaction was significant, supporting the notion that these variables are affecting the orthographic lexical system; (2) *INST + WT*—these two variables showed an additive pattern on RT, supporting the notion that they are affecting separable systems, namely the orthographic lexical system and the phonological output system, respectively; (3) *INST × SND*—the over-additive INST × SND interaction was significant, supporting the idea that the orthographic lexical system is affected by both variables; (4) *SND × WT*—the over-additive SND × WT interaction was also significant, which is congruent with SND and WT both affecting the phonological output system; (5) *WF × WT*—the WF × WT over-additive interaction under the normal *name all* INST was significant, supporting the notion that these variables affect the phonological output system.

Our RD analyses showed a similar pattern as Experiment 1. Consistent with Experiment 1, there was a main effect of WT whereby RD is shorter for the lexically read EXCs (*EXC RD < REG RD*), and approaches significance for INST (*name words RD < name all RD*), further supporting our hypotheses about shorter RDs being associated with lexically read items. Larger WF effects were again associated with the longer RD cells (i.e., REGs), despite the fact that the opposite pattern was demonstrated for onset RT. Our analysis of the SND effect on RD showed no significant effects.

The consistency in the results between Experiment 1 and 2 is reassuring, given that a voice-key was used to collect onset RT in Experiment 2, whereas hand-marking of onset RT was used in Experiment 1 (see Figures 6 and 8; cf. Rastle and Davis, 2002). Given that Experiment 1 was conducted in a MRI, we could not use a voice-key, but the gradient noise from the MRI scanner served as an effective auditory cue for identifying stimulus onset on the recording in that it was synchronized to appear co-incidently with the last image acquisition prior to the gap for responding. In Experiment 2, we used a voice-key for detecting onset RT as we did not include an auditory cue for stimulus onset.

## Analysis of combined Experiments 1 and 2 data

### Word naming reaction time

**Figure 11:**
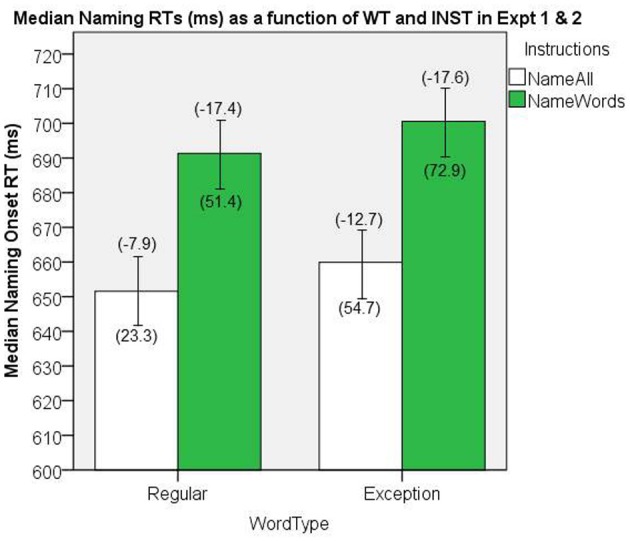
**Median Naming RTs (in ms) as a function of WT and INST in the combined analysis of Experiments 1 and 2.** The 95% C.I.s are presented as error bars using Loftus and Masson, (1994) method. Co-efficients relating WF to RT (ms/log_10_HALWF) are presented in parentheses above each error bar, and co-efficients relating SND to RT (ms/unit inverseNcount) are presented in parentheses below each error bar.

The data from the two experiments were combined, and the results were analyzed for all participants together (*n* = 59). The median naming onset RTs are presented in Figure 11. There were significant main effects of INST, [*F*_(1, 58)_ = 31.7, *MS*e = 3009.1, *p* < 0.001], and WT, [*F*_(1, 58)_ = 8.6, *MS*e = 528.2, *p* = 0.005], and no significant interaction, [*F*_(1, 58)_ = 0.03, *MS*e = 303.5, *p* = 0.85].

### Non-word naming reaction time

The NW condition in the *name all* INST condition yielded a median onset RT of 729.4 ms (Loftus and Masson, 1994, repeated-measure 95% CI = ± 12.1).

### Accuracy

Given that there was no variance in Experiment 1 accuracy data, we did not perform a combined analysis of the Experiments 1 and 2 data.

### Word frequency effects on reaction time

We conducted analyses of WF effects on RT in the same way as our analyses in Experiments 1 and 2. Figure 11 shows the mean co-efficients above the median RTs. There was a significant main effect of INST on the size of WF effect, [*F*_(1, 58)_ = 23.4, *MS*e = 129.2, *p* < 0.001], which represents an INST by WF over-additive interaction on naming RT, whereby the WF effects are greater under *name words* INST. The main effect of WT on the size of the WF effect approached significance, [*F*_(1, 58)_ = 3.18, *MS*e = 112.6, *p* = 0.08], which suggests a WT by WF over-additive interaction on naming RT averaging over both levels of the INST manipulation, whereby the WF effects are greater for EXCs than for REGs. More importantly, the analysis of this interaction under the normal *name all* INST condition yielded a significant effect, [*t*_(58)_ = −2.49, *SEM* = 1.91, *p* = 0.02]^2^

### Semantic neighborhood density effects on reaction time

We conducted analyses of SND effects on RT in the same way as our analyses in Experiments 1 and 2. Figure 11 shows the mean co-efficients below the median RTs. There was a significant main effect of INST on the size of the SND effect, [*F*_(1, 58)_ = 10.08, *MS*e = 3134.9, *p* = 0.002], which represents an INST by SND interaction on naming RT, whereby the SND co-efficients are greater in the *name words* INST condition than in the *name all* condition. There was also a significant main effect of WT on the size of the SND effect, [*F*_(1, 58)_ = 9.70, *MS*e = 4264.6, *p* = 0.003], which represents a SND by WT interaction on naming RT, whereby the SND co-efficients are greater for EXCs than for REGs.

### Word frequency and semantic neighborhood density joint effects on reaction time

A GLM regression was conducted on each participant's correct onset RTs, with RT as the dependent variable, and SND and WF as continuous independent variables, separately for each combination of INST and WT. Given that this constitutes a multiple regression, we note that, here and in later multiple regression analyses, the issue of multicolinearity was handled by using a tolerance threshold set at 0.0001, and there were no situations whereby this threshold was exceeded. Multivariate outliers were assessed using Malhalanobis distance, and there were no multivariate outliers exceeding the threshold of [χ^2^_(2)_ = 13.816, *p* < 0.001]. The resulting WF × SND co-efficients for each INST and WT set were then tested against zero using one-sample *t*-tests. The WF × SND co-efficients were significantly different from zero for REGs in the *name all* INST condition, [*t*_(58)_ = −2.40, *SEM* = 7.96, *p* = 0.02], and for REGs in the *name words* INST condition, [*t*_(58)_ = −2.11, *SEM* = 8.46, *p* = 0.04]. The WF × SND co-efficients for EXCs in the *name words* INST condition approached significance, [*t*_(58)_ = −1.77, *SEM* = 8.65, *p* = 0.08], and the co-efficients for EXCs in the *name all* INST condition were not significant, [*t*_(58)_ = −1.21, *SEM* = 5.76, *p* = 0.23].

### Word naming response duration

**Figure 12:**
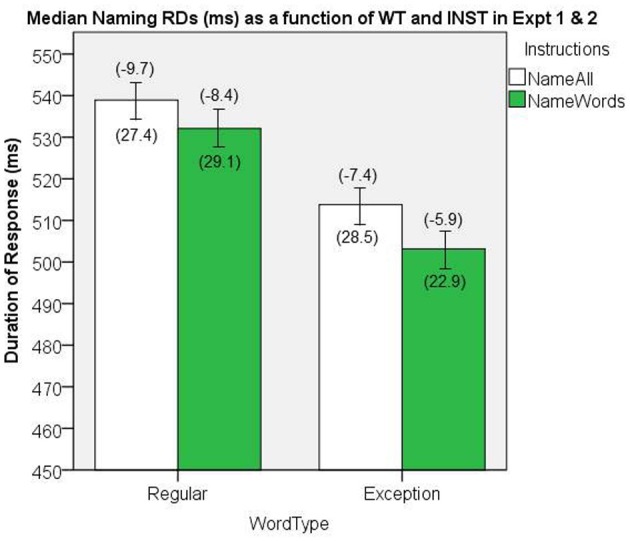
**Median Naming RDs (in ms) as a function of WT and INST in the combined analysis of Experiments 1 and 2.** The 95% C.I.s are presented as error bars using Loftus and Masson, (1994) method. Co-efficients relating WF to RT (ms/log_10_HALWF) are presented in parentheses above each error bar, and co-efficients relating SND to RT (ms/unit inverseNcount) are presented in parentheses below each error bar.

The median naming RDs are presented in Figure 12. There was a significant main effect of WT, [*F*_(1, 58)_ = 257.6, *MS*e = 167.7, *p* < 0.001], and of INST, [*F*_(1, 58)_ = 4.39, *MS*e = 1023.9, *p* = 0.04], and no significant interaction, [*F*_(1, 58)_ = 1.39, *MS*e = 156.6, *p* = 0.24].

### Non-word naming response duration

The NW condition in the *name all* INST condition yielded a median RD of 570.4 ms (Loftus and Masson, 1994, repeated-measure 95% CI = ± 6.72).

### Word frequency effects on response duration

We conducted analyses of WF effects on RD in the same way as our analyses in Experiments 1 and 2. The main effect of INST on the size of WF effect approached significance, [*F*_(1, 58)_ = 3.55, *MS*e = 34.7, *p* = 0.065], which suggests a INST by WF interaction on naming RD, whereby the WF effects are greater for the *name all* INST condition. The main effect of WT on the size of WF effect was significant, [*F*_(1, 58)_ = 9.77, *MS*e = 35.0, *p* = 0.003], which represents a WT by WF interaction on naming RD, whereby the WF effects are greater for REGs. Figure 12 shows the mean co-efficients above the median RDs.

### Semantic neighborhood density effects on response duration

In order to evaluate the effects of SND as a continuous variable, GLM regressions were conducted on each participant's correct RDs, as in Experiments 1 and 2. Figure 12 shows the mean co-efficients below the median RDs. There was no significant main effect of INST on the size of the SND effect, [*F*_(1, 58)_ = 0.145, *MS*e = 1429.3, *p* = 0.70], nor was there a significant main effect of WT on the size of the SND effect, [*F*_(1, 58)_ = 0.509, *MS*e = 742.5, *p* = 0.48].

### Word frequency and semantic neighborhood density joint effects on response duration

A GLM regression was conducted on each participant's correct onset RDs, with RD as the dependent variable, and SND and WF as continuous independent variables, separately for each combination of INST and WT. The resulting WF × SND co-efficients for each INST and WT set were then tested against zero using one-sample *t*-tests. The WF × SND co-efficients were significantly different from zero in the *name all* INST condition, for both EXCs, [*t*_(58)_ = −6.80, *SEM* = 2.74, *p* < 0.001], and REGs, [*t*_(58)_ = −3.12, *SEM* = 8.28, *p* = 0.003]. The WF × SND co-efficients for EXCs in the *name words* INST condition approached significance, [*t*_(58)_ = −1.94, *SEM* = 3.82, *p* = 0.057], and the co-efficients for REGs in the *name words* INST condition were not significant, [*t*_(58)_ = −1.13, *SEM* = 4.44, *p* = 0.26].

### Item analyses for reaction time

An item analysis was performed on the combined data from Experiments 1 and 2. Median onset RTs for both *name all* and *name words* INST were treated as repeated measure dependent variables, and regressed on WT and INST using a repeated measure GLM. Given that our measure of WF was moderately correlated with our measure of SND, *r* = −0.674 when we use the inverse Ncount measure, as we have in these analyses (*r* = 0.861 when the non-inverse Ncount measure is used), we chose to analyze the effects of WF and semantic density in separate regression models, as well as together in a subsequent model so that we could assess all of the Two-Way joint effects.

In the regression model that included WF but not SND, there was a significant main effect of INST, [*F*_(1, 248)_ = 113.38, *MS*e = 2057.9, *p* < 0.001], and a main effect of WT, [*F*_(1, 248)_ = 4.53, *MS*e = 3896.8, *p* = 0.03]. There was also a significant main effect of WF, [*F*_(1, 248)_ = 104.33, *MS*e = 3896.8, *p* < 0.001]. There was no significant interaction between INST and WT, [*F*_(1, 248)_ = 0.289, *MS*e = 2057.9, *p* = 0.59]. There was a significant INST by WF interaction, [*F*_(1, 248)_ = 50.05, *MS*e = 2057.9, *p* < 0.001]. The WT by WF interaction approached significance by a one-tailed test, [*F*_(1, 248)_ = 2.53, *MS*e = 3896.8, *p* = 0.057].

In the regression that included SND but not WF, there was a significant main effect of INST, [*F*_(1, 248)_ = 118.50, *MS*e = 2176.0, *p* < 0.001]. There was no significant main effect of WT, [*F*_(1, 248)_ = 0.27, *MS*e = 4562.7, *p* = 0.60]. There was a significant main effect of SND, [*F*_(1, 248)_ = 51.45, *MS*e = 4562.7, *p* < 0.001]. There was no significant interaction between INST and WT, [*F*_(1, 248)_ = 1.24, *MS*e = 2176.0, *p* = 0.26]. There was a significant interaction between INST and SND, [*F*_(1, 248)_ = 34.0, *MS*e = 2176.0, *p* < 0.001]. There was a significant WT by SND interaction, [*F*_(1, 248)_ = 4.13, *MS*e = 4562.7, *p* = 0.043].

In the regression that included both WF and SND, there was a significant main effect of INST, [*F*_(1, 245)_ = 24.89, *MS*e = 2034.2, *p* < 0.001]. There was no main effect of WT, [*F*_(1, 245)_= 0.01, *MS*e = 3757.9, *p* = 0.94]. There was a significant main effect of SND, [*F*_(1, 245)_ = 9.38, *MS*e = 3757.9, *p* = 0.002], and a significant main effect of WF, [*F*_(1, 245)_ = 27.83, *MS*e = 3757.9, *p* < 0.001]. There was no significant interaction between INST and WT, [*F*_(1, 245)_ = 0.01, *MS*e = 2034.2, *p* = 0.93]. There was a significant interaction between INST and SND, [*F*_(1, 245)_ = 4.23, *MS*e = 2034.2, *p* = 0.041]. There was a significant interaction between INST and WF, [*F*_(1, 245)_ = 10.01, *MS*e = 2034.2, *p* = 0.002]. The WT by SND interaction approached significance, [*F*_(1, 245)_ = 3.43, *MS*e = 3757.9, *p* = 0.065] (which, given the significant interaction in our earlier analyses, could be assessed by a one-tailed test with *p* = 0.0325). There was no significant WT by WF interaction, [*F*_(1, 245)_ = 0.01, *MS*e = 3757.9, *p* = 0.93]. There was a significant interaction between SND and WF, [*F*_(1, 245)_ = 8.24, *MS*e = 3757.9, *p* = 0.004].

### Item analyses for response duration

An item analysis was performed on the combined data from Experiments 1 and 2. Median RDs for both *name all* and *name words* INST were treated as repeated measure dependent variables, and regressed on WT and INST using a repeated measures GLM.

In the regression model that included WF but not SND, there was a significant main effect of WF, [*F*_(1, 248)_ = 20.9, *MS*e = 5249.7, *p* < 0.001]. There were no other significant main effects or interactions.

In the regression model that included SND but not WF, there was a significant main effect of INST, [*F*_(1, 248)_ = 9.21, *MS*e = 1043.1, *p* = 0.003], a significant main effect of WT, [*F*_(1, 248)_ = 7.21, *MS*e = 5518.0, *p* = 0.008], and a significant main effect of SND, [*F*_(1, 248)_ = 7.83, *MS*e = 5518.0, *p* = 0.006]. There were no significant Two-Way interactions.

In the regression that included both WF and SND, the only significant main effect was WF, [*F*_(1, 245)_ = 6.75, *MS*e = 5230.4, *p* = 0.01], and the main effect of SND approached significance, [*F*_(1, 245)_ = 3.10, *MS*e = 5230.4, *p* = 0.08]. The only Two-Way interaction that approached significance was between SND and WF, [*F*_(1, 245)_ = 3.56, *MS*e = 5230.4, *p* = 0.06]. There were no other significant main effects or interactions.

## General discussion

As described in the introduction, our first set of hypotheses involved the joint effects of INST, WF, SND, and WT on onset RT. Taken together, the by-participants analyses, the by-item-by-participant regression analyses, and the by-item analyses, support our hypotheses such that: (1) *INST × WF*—the INST × WF over-additive interaction was significant, supporting the notion that these two variables are affecting the orthographic lexical system; (2) *INST + WT*—given that INST should be having its effect in the early stages of word processing, whereas WT has previously been shown to have its effect at a later stage of processing, this additive pattern was also consistent with our previous research (Cummine et al., 2010, 2012; Borowsky et al., 2012), and supports the notion that INST are affecting an orthographic lexical system that is temporally separable from the phonological output system that is affected by WT; (3) *INST × SND*—the INST × SND over-additive interaction was significant, supporting a common locus of the orthographic lexical system for their effects; (4) *WF × SND*—the WF × SND over-additive interaction supports the notion that these two variables are affecting the semantic system and the connections to other word-level systems; (5) *SND × WT*—the SND × WT over-additive interaction was significant, supporting a common locus of the phonological output system for their effects; and (6) *WF × WT*—the WF × WT over-additive interaction under the normal *name all* INST was significant in Experiment 2 and the combined analyses, supporting the notion that these variables affect the phonological output system.

Our second set of hypotheses involved the RDs of vocalizations: *EXC RD* < *REG RD < NW RD*—given that EXCs must be processed as whole-words and read lexically in order to be pronounced correctly, they produced the shortest RDs, despite the fact that EXC onset RTs are longer than REG onset RTs. Given that NWs must be processed through sublexical GPCs, they produced the longest RDs. Finally, given that REGs can be processed through either route, they elicited intermediate RDs relative to EXCs and NWs, despite having the fastest onset RTs. The results supported the prediction that *name words RD < name all RD* in that the more lexically a word is read, the shorter the RD. The SND effect remained in the same facilitative direction for onset RT and RD, which is consistent with Balota et al. (1989) finding with semantic priming. The WF effect also remained in the same facilitative direction for onset RT and RD, which is consistent with it having its effects at the same lexical/semantic level as SND.

## Response duration

**Figure 13:**
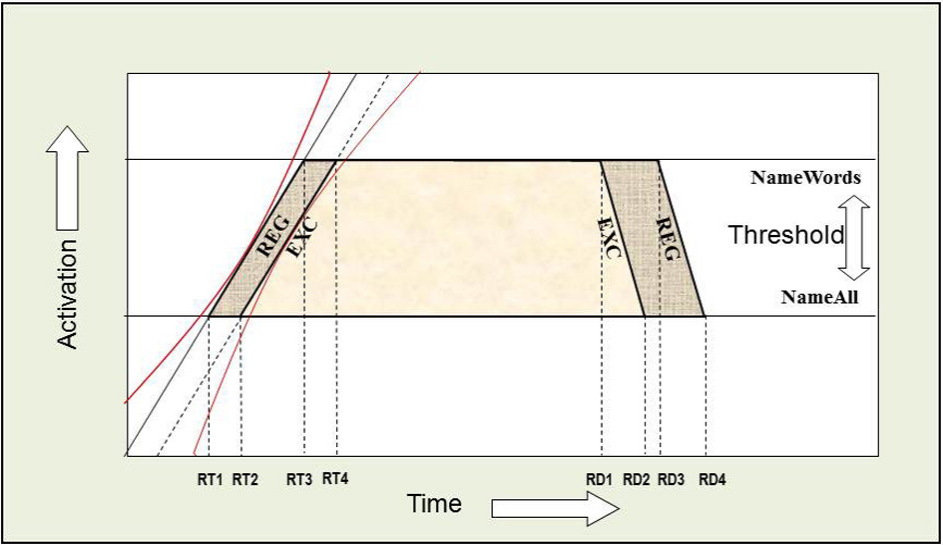
**Additive joint effects on RT and RD between INST and WT.** An Additive Factors Method (AFM) interpretation of the additive effect on RT is that INST and WT are affecting separable systems in time. If INST are assumed to affect the threshold for activation (i.e., the amount of time it takes to verify that a letter string spells a word), and WT is assumed to shift the rate of activation over time (i.e., the time it takes to choose among the competing phonological codes for EXCs), or vice versa, then the points in time when each rate crosses a threshold correspond to the average onset RTs (the left side of each trapezoid). The red confidence intervals around the slopes allow for trial to trial variation as described by Masson and Kliegl (2012), and thus we note that such variation is not problematic for applying the AFM, and can be easily accommodated by cascaded stages of processing. The effects of INST and WT on RD represent a dissociation when compared to RT. The right side of each trapezoid represents the RD and illustrates the dissociation.

**Figure 14:**
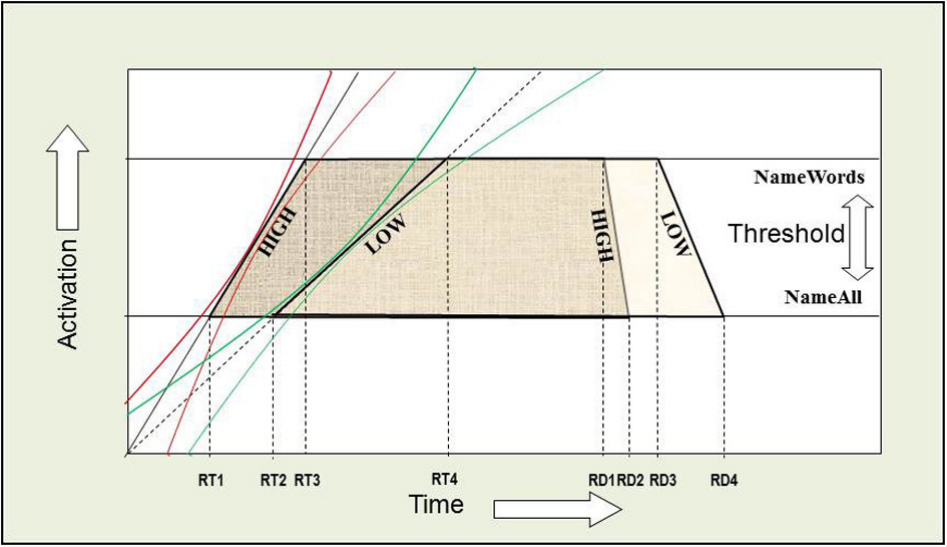
**Over-additive interaction on RT between INST and WF.** An AFM interpretation of this interaction on RT is that both INST and WF are affecting a common system in time. If INST are assumed to affect the threshold for activation and WF is assumed to affect the rate of activation over time, or vice versa, then the points in time when each rate crosses a threshold correspond to the average onset RTs (the left side of each trapezoid). WF and WT interact on RT in a similar fashion. The red and green confidence intervals around the slopes allow for trial to trial variation. The effects of WF on RD remain negative when compared to RT. We note that SND effects can be accounted for in a similar manner. The effects of INST on RD represent the dissociation when compared to RT. The right side of each trapezoid represents the RD and illustrates this dissociation.

By developing a new measure of RD for reading aloud, we have an additional and more comprehensive measure of basic reading processes. Given that basic reading processes are still ongoing after the initiation of a vocal response, measures of onset RT may only reflect early aspects of processing (e.g., in terms of only *partially* reflecting lexical access, or the resolution of conflicting phonological codes). Our results provide evidence that systems that are influenced by such variables as INST, WF, SND, and WT are still affecting the duration of the reading response, even after these variables have already influenced onset RTs. In addition, our results support the notion that the more lexically a word is read (e.g., EXCs, or *name words* INST), the shorter the RD, in the face of longer onset RTs for such conditions (see Figures 13 and 14). To our knowledge, this is the first demonstration of dissociation between RT and RD, as a function of the degree of lexical-based reading. This dissociation is particularly powerful given that it has been demonstrated by both a within-item (i.e., INST) and between-item (i.e., WT) manipulation. Although Balota et al. (1989) showed that semantic priming had an effect on both RT and RD in their study, whereby both were shorter for the related condition, WT and INST in the present study are clearly showing a dissociation between onset RT and RD. Interestingly, neither SND nor WF effects reversed between RT and RD (i.e., inverse Ncount co-efficients remained positive, while WF co-efficients remained negative in all conditions), and thus seem to be behaving in a manner consistent with semantic priming effects (Figure 14).

Perhaps SND and WF effects (similar to Balota et al. (1989) finding with semantic priming) can be thought of as consistently reflecting the core lexical/semantic aspects of processing, in that they both facilitate the speed of lexical/semantic access and thus affect onset RT and RD in the same way. Instruction effects might best be thought of as reflecting a front-end gating manipulation, whereby INST to *name words* serves to increase reliance on the orthographic lexical route, relative to INST to *name all*. RT is higher for *name words* INST given the time required to verify the word's lexical status, whereas RD is shorter in this condition given that once the word's lexical status has been verified, the phonological code is also lexical-based and thus more rapidly produced. However, it is clear that EXCs are showing different RDs for the two INST conditions, suggesting that there is “room to move” for EXCs to have even shorter RDs under the *name words* INST condition compared to the *name all* INST condition. Perhaps under *name all* INST, EXC RDs are produced with some hesitation due to the greater overall reliance on sublexical GPCs under *name all* INST (e.g., for naming the NWs, and perhaps REGs some of the time). Word-type effects might best be thought to reflect a back-end convergence effect, whereby both routes produce converging phonological codes for REGs, relative to EXCs whereby both routes produce conflicting phonological codes. RT is shorter for REGs given an early assessment of the phonological codes for the word's onset, allowing a participant to quickly initiate their response, whereas RD is longer given the slower sublexical contribution to completing the entire word's pronunciation.

The present RD results also bear on the question of the degree to which reading processes are still occurring post-vocalization onset. Given the significant effects of INST, WF, SND, and WT on RD, there is clearly a substantial amount of processing occurring post-vocalization onset. These post-vocalization onset effects clearly support the utility of an RD measure for investigating reading processes.

## Additive factors method and models of reading

The AFM allows for the investigation of whether two or more variables are affecting common or separable systems in time, whereby two variables that interact over-additively on RT are considered to affect a common system, whereas two variables that are additive on RT are considered to affect separable systems. We selected four lexical/semantic variables known to affect basic reading processes, and examined their joint effects so as to delineate the sequence of systems involved in reading aloud. We manipulated: INST as a variable that would serve to gate processing toward the lexical route when participants were to *name words* only; WF as a variable whose effects reflect lexical/semantic connections; SND as a semantic variable whose effects reflect associations among semantic neighbors and their lexical/semantic connections; and WT as a variable whose effects reflect the convergence of the sublexical and lexical routes, in that REGs can be pronounced correctly through either route, whereas EXCs create conflicting phonological codes through the two routes. The INST × WF and INST × SND over-additive interactions support the notion that these variables are affecting a common and relatively early system in time, namely the orthographic lexical system (see Figure 1). The WF × WT and SND × WT over-additive interactions support the notion that these variables affect a common and relatively later system in time, namely the phonological output system. The INST + WT additive joint effects support the notion that they are affecting the orthographic lexical and phonological output systems, respectively. Taken together, these joint effects clearly support a model where the orthographic lexical system and phonological output system are cascaded in time (Figure 1), and not operating in parallel (cf., Seidenberg et al., 1996; Plaut and Booth, 2000).

The issue of the naming task being less sensitive to semantic effects, as described in the Introduction (Borowsky and Masson, 1996; Yap et al., 2012), is also addressed with the present results. By instructing readers to pronounce words only after lexical verification (i.e., the *name words* INST condition), and thus requiring them to read lexically, there was a larger SND effect than when they were instructed to name without encouraging reliance on the lexical route (i.e., the *name all* INST condition). As such, the INST × SND over-additive interaction supports the notion that semantic influences on naming behavior can occur under conditions that encourage lexical access. The SND × WT over-additive interaction also supports this notion, whereby EXCs, which must be processed via the orthographic lexical route, showed a larger SND effect than REGs^3^.

### Ventral-lexical, dorsal-sublexical, model of basic reading processes

Our preferred cognitive architecture for basic reading processes, which is based on the Dual-Route Cascade model (Coltheart et al., 2001), assumes that processing operates on two routes: a sublexical GPC route, which allows less familiar letter strings (including NWs and novel words) to be “sounded out,” and a lexical route, which allows familiar words to be read as whole-words (see Figure 1). These routes have been mapped onto the dorsal and ventral visual processing streams, respectively (Herbster et al., 1997; Jobard et al., 2003; Price and Devlin, 2003; Indefrey and Levelt, 2004; Joubert et al., 2004; Borowsky et al., 2006, 2007, 2012; Hickok and Poeppel, 2007; Cohen et al., 2008; Cummine et al., 2010, 2012). This model also assumes that processing is cascaded among the subsystems.

The ventral-lexical route is relied on for reading familiar REGs and EXCs. The dorsal-sublexical route is relied on for reading NWs, novel words, and less familiar REGs. The convergence of these two routes can be facilitative in the case of reading REGs (where the phonological codes would be the same from both routes), or conflicting in the case of reading EXCs (where the phonological codes would be different from both routes), which has been described earlier in the context of the interaction between WF and WT (see also Cummine et al., 2010). Given that WF and SND have their effects in the lexical/semantic systems, including the orthographic lexical system, their influences are early enough to interact with the effects of INST, which can gate processing through the lexical route under the *name words* condition. In order to allow for novel words that lack any lexical representation to be read aloud, there is also a pathway from GPC to phonological output (see also Borowsky et al., 2002).

It is worth noting that the ventral-lexical, dorsal-sublexical, multiple stage model and the effects of INST and WT that we are describing here are also consistent with other findings in the literature that have underscored the necessity of multiple stages and attentional control in basic reading models. For example, Reynolds and Besner (2005) showed that participants took longer to name both words and NWs when the item on the preceding trial was from the other lexical category, relative to when the preceding item was from the same lexical category, which is similar to our account for why EXCs show different RDs under the two INST conditions (see also Reynolds and Besner, 2006, for a multiple stage account of attention and reading processes). Furthermore, Reynolds and Besner (2011) have also showed changes in the WF effects as a function of list context when reading pseudohomophones aloud (see also Borowsky et al., 2002).

### Single-mechanism models

Single-mechanism parallel distributed processing (PDP) models (e.g., Plaut and Booth, 2000) are challenged by the current results. Such models have been developed to account for the basic effect of WT (REG, EXC), and the WF by WT interaction, on RT by a “division-of-labor” between an Orthography–Phonology (O–P) pathway and an Orthography–Semantics–Phonology (O–S–P) pathway (e.g., Harm and Seidenberg, 2004, although “pathway” may be misleading given that these models subscribe to parallel processing across the entire network). Larger WF effects for EXCs are thought to occur due to the additional WF-sensitive connections involved in the O–S–P pathway, compared to the O–P pathway that REG reading is thought to rely on. There is no distinct orthographic lexicon in these models, unlike the dual-stream models, and so INST to read by first checking the orthographic lexicon (name words, based on spelling) raises a challenge in and of itself. Waiting (to any degree) for the O units to settle on the word's pattern of activation and using that information to gate processing in the S and P units might be a solution, but such “stage-like” or “cascaded” processing is counter to the *parallel* definition of these models (Plaut and Booth, 2000; and see the debate by Borowsky and Besner, 2006; Plaut and Booth, 2006, and Besner and Borowsky, 2006, for additional discussion of these issues, and see Ziegler et al., 2009, for a more recent hybrid computational model that has implemented thresholds or “stages of processing” in order to account for some additive effects). Perhaps most challenging is the presence of additive effects on RT (i.e., INST and WT) in the same range of RTs that also show over-additive interactions. Although a sigmoid activation function within a single-mechanism PDP model has been explored as a means to account for both additivity and over-additive interactions (Plaut and Booth, 2000), this approach is problematic as additive effects can only arise equidistant from the center of the sigmoid input–output function, yet additive effects occur regularly within the very same range of RTs as over-additive effects, as demonstrated in the research reported here, and elsewhere (see Borowsky and Besner, 2006 and Cummine et al., 2012 for a review).

Additive effects in the same range of RTs as over-additive effects are still best accounted for by the AFM. Additive effects of two variables are easily accounted for by implementing the effects of the two variables at two different time points in processing (i.e., two systems with stage-like processing). These systems may be in cascade (e.g., McClelland and Rumelhart, 1981; Borowsky and Besner, 1993; Coltheart et al., 2001)—all that is necessary is at least some delay between the initiation of activation in one system compared to the other. Such a delay is parsimonious with the known behavior of real neural networks, and thus it can also be argued to be a necessary characteristic in all neurobiological models. Over-additive effects of two variables can be accounted for by implementing the effects of the two variables within the same system of processing (e.g., by affecting its activation rate, threshold, or baseline activation level, see Borowsky and Besner, 1993, 2006, for discussion about how these parameters can be modeled to account for additivity and over-additivity). Dual-stream models of reading can readily handle the over-additive interactions as long as cascaded processing (i.e., some degree of delay of activation in systems down-stream) is assumed.

### Advantages of the present method

Despite the amount of time that goes into hand-marking each vocalization, the benefits from this approach far outweigh the costs (see also Rastle and Davis, 2002). Not only does hand-marking provide a new set of empirical data (RD) for testing models of reading processes, it also provides the following advantages. The traditional definition of a spoiled trial in a naming experiment includes a substantial proportion of trials when the voice-key failed to trigger—in the present experiments, the proportion of spoiled trials is quite low, given that replaying the audio is an important part of zeroing-in on the onset and offset, which is not done when one relies on a voice-key. Recording and hand-marking of vocal responses also allows for the collection of overt naming behavioral data in the MRI environment. Experiment 1 was conducted in the context of a fMRI study, and by simply recording through the intercom and synchronizing stimulus onset with an image acquisition, we were able to clearly detect onset and durations of vocal responses. By also using sparse sampling (i.e., a gap in image acquisition), the participants' vocal response was made in a relatively noise-free time period, which was also helpful. As such, recording and hand-marking of vocal responses will be of great benefit to researchers who do fMRI experiments involving vocalization responses. We note that there has been some computer software developed to analyze for onset and duration (e.g., Kello and Kawamoto, 1998), but such an approach would not be as effective as hand-marking and replaying vocal responses with respect to detecting spoiled responses and individual differences in intensity of vocalization, especially in noisy environments such as an MRI.

## Conclusion

Pursuing an understanding of the meanings of things in our world is a central feature of the human condition. We have an insatiable curiosity to understand how to interact with objects in our environment, how to interpret symbols and actions, as well as the meanings of words. Given that semantic knowledge is core to understanding words, our present research explored the interactions between semantic and lexical variables in order to inform the development of a model of basic reading processes. The joint effects of INST, WF, SND, and WT on naming RT support a cascaded, dual-route, ventral-lexical/dorsal-sublexical model. Our naming RD results provide evidence that basic reading processes, and their joint effects, are occurring even after the initiation of a vocal response, and support the notion that the more lexically a word is read, the shorter the RD. Given the joint effects on RT, the dissociating effects of INST and WT on RT versus RD, and consistent effects of WF and SND on RT and RD, models of basic reading processes now have new challenges to accommodate these effects. An important question for future research is the degree to which RD effects are due to phonological-lexical vs. orthographic-lexical processing, which our lab is beginning to explore.

### Conflict of interest statement

The authors declare that the research was conducted in the absence of any commercial or financial relationships that could be construed as a potential conflict of interest.

## Footnotes

^1^Following the interpretation of the WF × WT interaction within a dual-route framework (e.g., Coltheart et al., 2001) the initiation of a response is modulated by the consistency in the computed phonological codes from sublexical and lexical routes. On one hand, in the case of EXCs, while the onset RT may be delayed due to the competing phonological codes, the RD should be relatively short given that there is greater reliance on the lexical system. Regular words, on the other hand, have a relatively fast onset RT because there is no competition from the sublexical and lexical routes. However, information from both the sublexical and lexical systems can contribute to correct responding and thus the RD may be longer to accommodate the inclusion of the sublexical information.

^2^We are concentrating on Two-Way interactions in this research given the focus on the Additive Factors Method. A reviewer had pointed out an interesting potential three-way interaction whereby the WF × WT interaction was only significant in the *name all* condition, but not the *name words* condition. Given that the WF effects are consistently negative for all of the conditions, any such Three-Way interactions would be ordinal (i.e., not a cross-over interaction), which are notoriously difficult to detect (i.e., all effects are in the same general direction). Nonetheless, we did examine tests for Three-Way interactions under the conditions in our study that had the most power to detect such interactions. We tested the WF × WT × INST interaction on RT, and it yielded the following result, [*F*(1, 38) = 1.67, *MS*e = 160.40, *p* = 0.204]. We also examined the corresponding interaction in the item analyses, and also found a non-significant result, [*F*(1, 248) = 0.43, *MS*e = 2057.89, *p* = 0.51].

^3^In response to a reviewer's query about SND effects under the *name all* INST condition, we conducted one-sample *t*-tests on the co-efficients that relate SND to RT and to RD. All SND co-efficients were found to be significantly different from zero (all *t*_(58)_ > 4.25, *p* < 0.001). We also examined WF effects in the same way, and found that all WF co-efficients were significantly different from zero (all *t*_(58)_ > |−7.06|, *p* < 0.001). These results support the previously described idea that both SND and WF effects consistently reflect the core lexical/semantic aspects of processing, in that they both facilitate the speed of lexical/semantic access and thus affect onset RT and RD in the same way.
